# Supplementary material for: Population structure of Neisseria gonorrhoeae based on whole genome data and its relationship with antibiotic resistance
Source: PeerJ. 2015 Mar 5;3:e806. doi: 10.7717/peerj.806 (PMC4358642; doi:10.7717/peerj.806)
Supplement: Supplemental Information 1 [file peerj-03-806-s001.pdf]

Supplemental Text

Protocol for searching for known mutations

The resultant blast output was parsed for resistance genes mentioned in the ontology to confer antibiotic resistance, using our custom python script *blast_read.py.* We also trimmed the output file removing resistance genes that are in the core genome or present in all the strains, so we ended up with flexible genes which are present in only a few strains but have similarity with known resistance genes in other species which could have been horizontally transferred to the respective *N. gonorrhoeae* strains.

I grouped the strains in our sample set based on their antibiotics resistance phenotypes, for each of the groups of antibiotics (macrolides, cephalosporins, tetracycline) we used in determining the MICs for the strains. Each group is further divided into the strains that have the known mutation mutations (determined through the bioinformatics alignment search) underlying resistance to the respective antibiotic and those that do not have such mutations.

We used the NCBI blast tools and command line prompts in Unix to create a nucleotide blast database. This database is a multi-sequence fasta file of the WGS shotgun sequences of all the strains in our sample set assembled using the velvetg assembler. The contigs for each assembly was ordered into one pseudo contig after tiling to the reference genome FA1090, using the abacas.pl perl script.

Next we obtained reference NCBI sequence data of genomic regions encompassing resistance variants that have be shown in the literature to underlie the resistance phenotype we have observed in our sample set, and we performed a blast search for each of these sequences across all the strains in our database.

We picked the top hit for each sequence (strain) in the database and parsed the alignment between the query and the subject sequence in the database for the presence or absence of the underlying resistance genetic mutations as suggested in the literature using our custom perl script *parse.pl*. The results of our bioinformatics searches are shown in Supplement Data S3.

A number of the strains that have reduced susceptibility to the antibiotics drugs in the clinical data set do not possess the expected mutations within their corresponding resistance loci as suggested in previous literature. These results, which are shown in Table 4 suggested the possibility of epistatic interactions between either known variant sites or between known variants and novel loci to give rise to the resistance phenotypes.

Pangenome Analysis

The OrthoMCL module basically performs an all by all BLAST query for all the genes across all the strains define in our sample set. Orthologs and paralogs are defined as the best match for a given gene in another strain or in the same strain respectively. The best matches are grouped in clusters representing similar genes from across the different strains and forming the pangenome.

The result from the analysis maps well with the expected distribution of the pan genome into the core and accessory genes. The extended core genes that comprises of genes that have homologues in each of the strains, generally represent genes that are functionally relevant to the species. The accessory genes on the other hand are unique to each strain. A plot of the distribution of the clusters of orthologous genes show that majority of the genes in the metagenome are either accessory or part of the extended core genes. See Fig S1."

Our custom scripts for this project are in the Github repository: https://github.com/Read-Lab-Confederation/Neisseria_gonorrhoea_Population_Study
