# Supplementary figures and images for: Population structure of Neisseria gonorrhoeae based on whole genome data and its relationship with antibiotic resistance
Source: PeerJ. 2015 Mar 5;3:e806. doi: 10.7717/peerj.806 (PMC4358642; doi:10.7717/peerj.806)

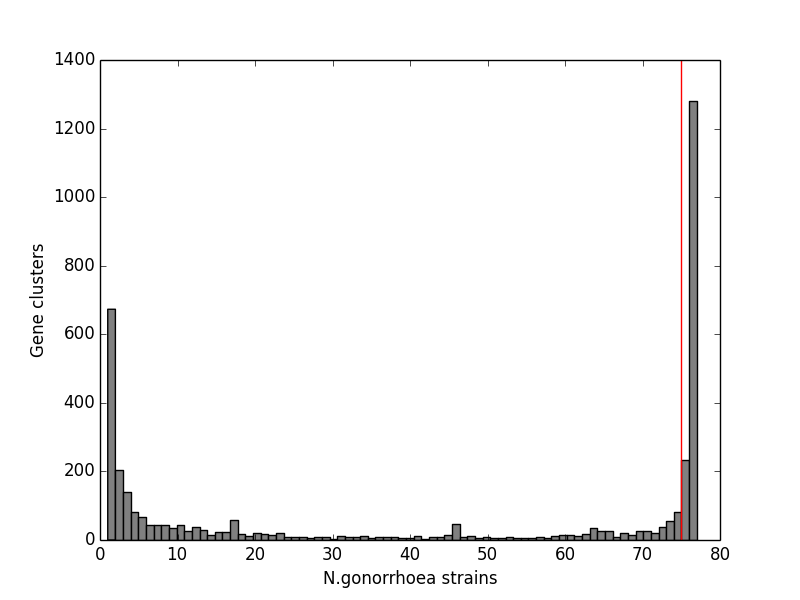

Supplement: Figure S1 — Each bar is a count of the number of genes (technically gene clusters) found in n genomes (n = 76). Area to the right of the red line represents the extended core genes; to the left are the non-core genes. [file peerj-03-806-s005.png]

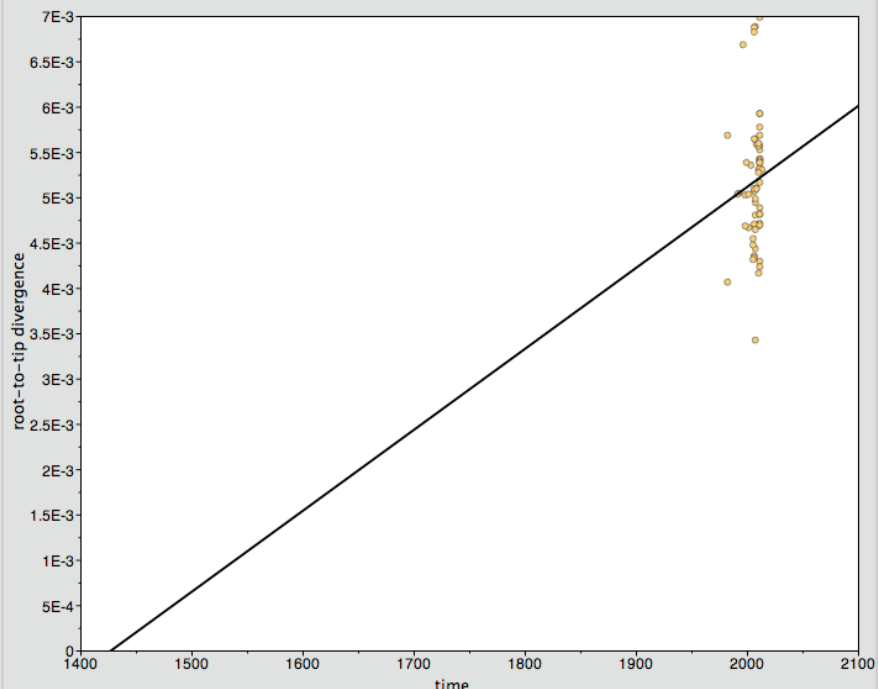

Supplement: Figure S2 [file peerj-03-806-s006.png]

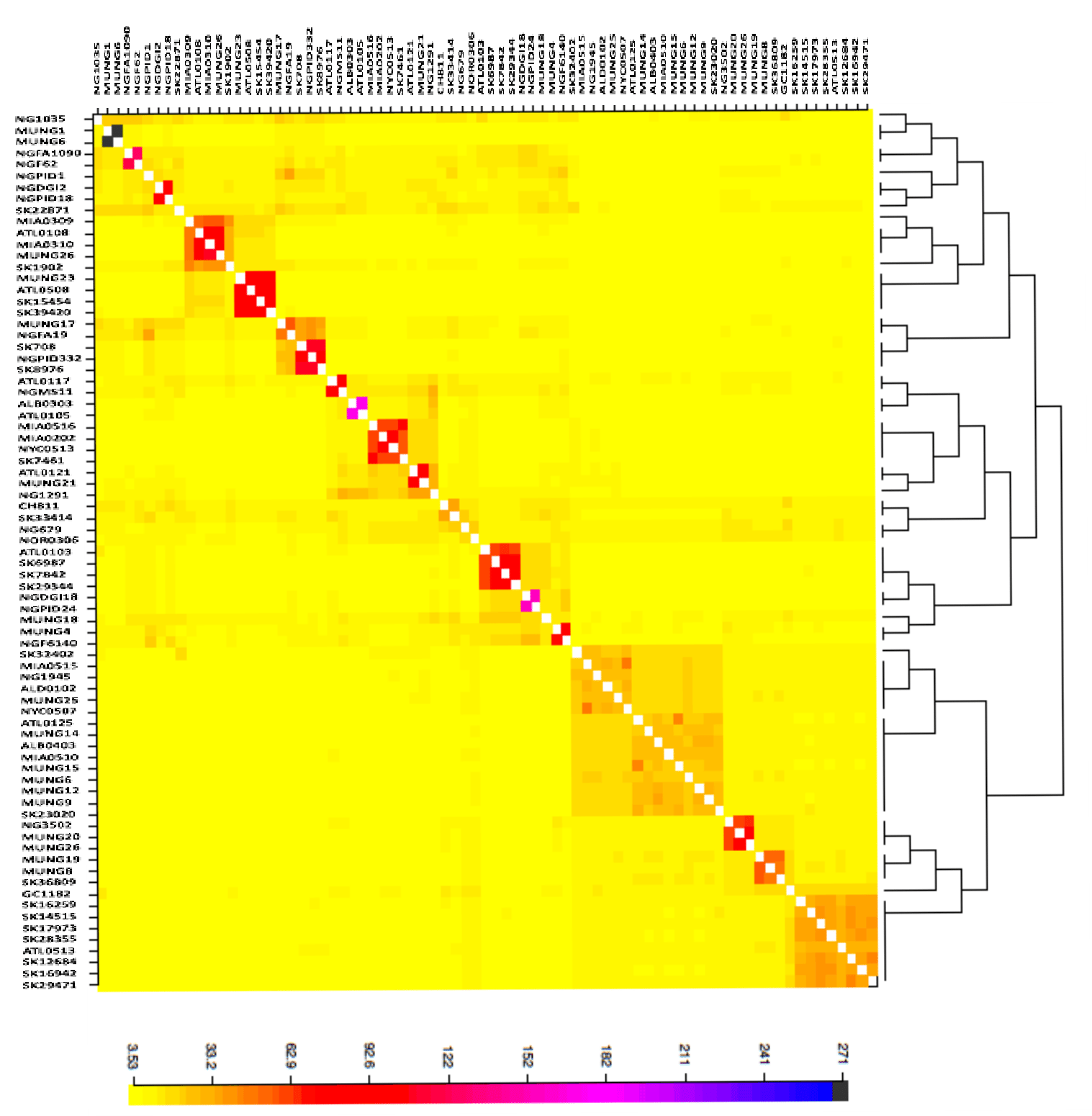

Supplement: Figure S4 — The figure was generated using fineSTRUCTURE tool, representing pairwise genetic relationship between the strains in the sample set. [file peerj-03-806-s008.png]

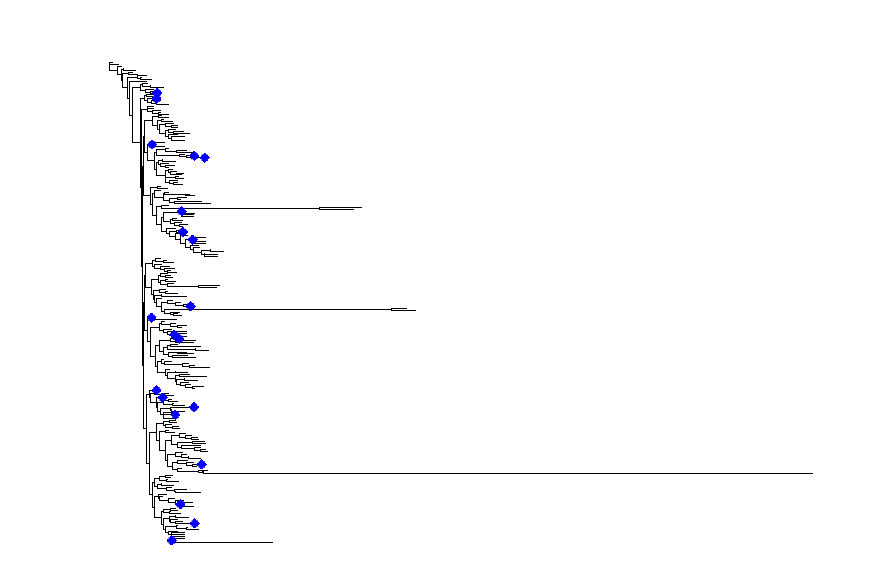

Supplement: Figure S5 — The blue colored spots represent majority of the sequence types present in our sample set. [file peerj-03-806-s009.png]
